# Structural and biological characterization of carbon–graphene biomaterials derived from black liquor with functional properties for bone tissue engineering

**DOI:** 10.1038/s41598-025-29606-x

**Published:** 2025-12-10

**Authors:** Patricia de Almeida-Mattos, Gustavo Lara Achôa, Danyela Cardoso Carvalho, Taro Inagaki, Marilia Lucas Siena Del-Bel, Daniel Navarro da Rocha, José Ricardo Muniz Ferreira, Mariah Cationi Hirata, Gisele Aparecida Amaral-Labat, Rodrigo Labat Marcos, Reza Jarrahy, Akishige Hokugo, Daniela Franco Bueno, Guilherme Frederico Bernardo Lenz e Silva

**Affiliations:** 1 Departamento de Engenharia Metalúrgica e de Materiais, Escola Politécnica da Universidade de São Paulo, São Paulo, Brasil; 2 Instituto Sírio-Libanês de Ensino e Pesquisa, São Paulo, Brasil; 3 Regenerative Bioengineering and Repair Laboratory, Department of Surgery, David Geffen School of Medicine, University of California in Los Angeles (UCLA), Los Angeles, USA; 4 Departamento de Biofotônica Aplicada às Ciências da Saúde, Universidade Nove de Julho (UNINOVE), São Paulo, Brasil; 5 Instituto Militar de Engenharia Mecânica - IME, Rio de Janeiro, Brasil; 6 Departamento de Bioengenharia, R-Crio Criogenia S.A, São Paulo, Brasil; 7 Instituto Nacional de Pesquisas Espaciais, INPE, São Paulo, Brasil; 8 Faculdade Israelita de Ciências da Saúde Albert Einstein, FICSAE, São Paulo, Brasil

**Keywords:** Tissue engineering, Bone repair, Carbon-graphene scaffold, Carbon related biomaterials, Biological techniques, Biotechnology, Stem cells, Medical research

## Abstract

Carbon-based biomaterials are promising for the field of tissue bioengineering due to their biocompatibility, high porosity, and physicochemical properties that allow functionalization and combination with other materials. In this study, carbon derived from black liquor was used to develop bone grafts. This carbon matrix (CA) was associated with nanomaterials—graphene, graphene oxide, and nano-graphite (CAG, CAGO, and CANG, respectively)—for potential applications in the repair of orofacial malformations. The scaffolds were evaluated for biocompatibility and their effect on cell viability using mesenchymal stem cells, followed by implantation in 16 male Wistar rats with non-critical bone defects in both tibias. Histological analysis demonstrated that all scaffolds were biocompatible, with defects showing repair, osteoprogenitor cell presence, and vascular channel formation. Histomorphometric assessment of bone neoformation revealed the highest repair potential in the CAG group (89% at 30 days), while the other groups showed similar bone formation: CA = 72%, CAGO = 69%, and CANG = 80%. All scaffolds promoted bone tissue formation, with the carbon-graphene scaffold yielding the greatest percentage of new bone.

## Introduction

Bone tissue engineering is a multidisciplinary field that involves the combination of biomaterials with cells or growth factors to promote new bone formation^1^. In this field, the development of bioactive scaffolds capable of inducing the formation of functional bone tissue is crucial^2^. Therefore, ideal scaffolds for bone tissue formation must have three-dimensional porous structures that are biocompatible and exhibit controlled degradation. These scaffolds should also be capable of incorporating bioactive elements that promote a localized response for bone tissue repair^3^. Moreover, it is important to highlight that a three-dimensional scaffold must possess interconnected pores to allow the passage of nutrients, oxygen, and metabolites, which are essential for vascularization and new bone tissue growth^4^.

Carbons have been utilized in the composition of scaffolds for bone tissue engineering due to their high porosity and rough surface. Furthermore, the use of carbon in scaffold fabrication offers several advantages, such as easy integration with other biomaterials and a nanostructure that enhances biological interaction with adjacent tissues. These characteristics are fundamental for cell adhesion and migration, thereby facilitating the formation of new bone tissue^5^. Various methods can be used to produce carbon, including the use of raw materials, resulting in a biomaterial that is both easily fabricated and cost-effective^6–9^.

Additionally, carbon possesses tunable microstructural characteristics that can be exploited to develop multifunctional biomaterials. Both two- and three-dimensional structures can be created from these materials, such as nanotubes, graphene, and graphene oxides. Furthermore, functional chemical groups present on the surface can be used to carry molecules^10,11^, proteins, growth factors^12^, and cells such as mesenchymal stem cells^10,13,14^. All of these properties of carbons have demonstrated positive effects in the use of these composites for bone repair.

The literature shows that a significant challenge in bone tissue engineering is controlling scaffold degradation and malleability, as the loss of structural support during bone development can critically affect proper defect repair. To address this, some polymeric materials have been combined with carbon and its derivatives to control these degradation and malleability properties^15,16^.

In medicine, controlling the degradation and malleability of biomaterial scaffolds is critical for bone tissue engineering, particularly in the rehabilitation of pathologies such as cleft lip and palate (CLP). For successful alveolar bone formation in CLP patients, it is essential to use scaffolds with regulated resorption to allow the eruption of the canine tooth into the newly formed alveolar bone.

It is important to note that cleft lip and palate (CLP) is the most common congenital facial anomaly, representing approximately 25% of all congenital defects^17^. In the quest to enhance bone repair interventions, studies have been conducted worldwide using growth factors, bioactive molecules, and mesenchymal stem cells^18,19^ in combination with synthetic biomaterials. These efforts aim to develop new bone tissue through tissue engineering strategies, reducing the morbidity and pain associated with harvesting autogenous bone for alveolar grafts.

In this context, our research group conducted a multicenter clinical study using autologous stem cells from the pulp of deciduous teeth combined with a biomaterial composed of collagen and hydroxyapatite to rehabilitate alveolar bone in patients with cleft lip and palate^20,21^. Although alveolar bone formation occurred in all participating patients, we observed that the biomaterial used in the rehabilitation was costly^22^ and that there were secondary issues such as the impaction of teeth near the graft region during eruption due to slow degradation^23^. When tooth impaction occurs, it is necessary to perform orthodontic traction of the tooth in the oral cavity, thereby increasing both the cost and duration of dental treatment. In our multicenter study^21^, we observed canine impaction in 30% of cases, highlighting the need to develop a biomaterial with faster controlled resorption and lower cost for use in alveolar bone tissue engineering.

Therefore, the objective of this work is to develop carbon-graphene-based biomaterials, combine them with a chitosan-xanthan-based polymeric compound, and evaluate the effectiveness of these biomaterials for bone formation, aiming at their future use in bone tissue engineering for patients with cleft lip and palate, due to their excellent structural properties.

## Materials and methods

This study is a partnership between our research group (LM_2_C_2_ - High Energy Milling, Carbon Materials and Composites for High Temperatures Laboratory) at the University of São Paulo, Sírio-Libanês Hospital, University of California Los Angeles (UCLA) and R-Crio Company, Campinas, SP. It was developed in two phases, the first consisted of developing the scaffolds (carbon composites associated to chitosan-xanthan compound) and second, the implantation of the final scaffolds (chitosan-xanthan + carbon composites) in an animal model.

The carbon composites were prepared according to their composition: (1) CA (pure carbon); (2) CAG (carbon associated with graphene); (3) CAGO ( pure carbon associated with reduced graphene oxide) and (4) CANG (carbon associated with nanographite). After carbon production, the scaffolds were prepared according to previous protocols developed at R-Crio Company.

The in vivo test protocols were analyzed and approved by the Research Ethics Committee of the Universidade Nove de Julho and were carried out according to recommendations of the National Animal Welfare Commission (COBEA).

### Carbon composites synthesis

A sustainable, low-cost, and highly porous carbon material was developed and combined with nanostructured carbons to create scaffolds for potential applications in tissue engineering. The primary precursor for the carbon matrix was raw Kraft black liquor, a by-product of the paper and pulp industry produced in large quantities.

Pure carbon matrix (CA) was obtained by chemical polymerization of black liquor (100 g, Suzano Papel e Celulose SA) and resorcinol (15 g, Sigma^®^) following the protocol described by Amaral-Labat et al.^24^ with adaptations. The reagents were mixed under stirring until the resorcinol was completely dissolved. After that, it was added 45 g of poly(methyl methacrylate) with granulometry of Ф ≤850 μm (Unigel^®^) and 44 g of formaldehyde (Synth^®^), both added to ensure controlled pore formation, viscosity increase and solidification.

The composites containing carbon and nano-derivatives (CAG, CANG and CAGO) were prepared following the same protocol, however, 0.1 wt% of each nanomaterial was added immediately before the solidification within 1 g of graphene – CAG; 1 g of nano-graphite – CANG (both provided by MG-graphene Project) and 1 g of Graphene Oxide – CAGO (modified Hummer’s method synthesis). After solidification, the samples were kept in a chemical safety hood to dry for 10 days.

After drying, the samples were heated in a tubular oven with a working temperature of 900 °C for 2 h (5 °C/min) under inert atmosphere (Argon). After, the samples were washed in Soxhlet Extraction System^25^ during 10 days, dried at 100 °C for 24 h and ground to ≥ 250 ≤ 450 μm. After that, all samples were sterilized (wet method) and dried again in an oven at 100 °C, overnight.

### Synthesis carbon composites scaffolds

At R-Crio laboratories, after preparing the carbon composites, scaffolds were fabricated by mixing chitosan-xanthan (QXA) polymers and carbon composites to promote 3D structure and malleability. For this, it was prepared the QXA compound following the protocol proposed by Neves et al. (2022)^26^ and Souza et al. (2022)^27^ with low-molecular-weight chitosan (Sigma-Aldrich^®^) solution at 1% (w/v) and xanthan gum (Sigma-Aldrich^®^) solution at 1% (w/v).

After mixing both solutions, QXA compound and powders of CA, CAG, CANG and CAGO were added in a 1:1 ratio (50 wt%-QXA; 50 wt%-carbon composites) and transferred to an acrylic cuvette (Fastlab) measuring 0.5 × 0.5 × 4 cm. The samples were dried at room temperature for 48 h and after that, placed in a freezer at -30 °C during 24 h. Then, the scaffolds were lyophilized for 48 h under vacuum (≤ 0.04 mbar) and were sterilized (ethylene oxide 30% and carbon oxide 70%) prior to in vitro and in vivo tests.

### Scaffolds microstructural characterization

Morphology analyzes were performed by scanning electron microscopy (SEM) using INSPECT™ F50 equipment (15 kV; FEI, Oregon, USA). The surface chemical composition was semiquantitatively analyzed by energy-dispersive X-ray spectroscopy (EDS) –EDAX™ detector (122 eV; Ametek, NJ, USA).

The specific surface area (*S*_BET_) was analyzed by nitrogen adsorption/desorption on ASAP 2020 Plus (Micromeritics, Georgia, USA) at − 196 °C. Samples were outgassed at 90 °C for 24 h. The data were calculated by Brunauer-Emmet-Teller (1938)^28^ method.

Fourier transform infrared spectroscopy (FTIR) (IRTracer-100; Shimadzu, Kyoto, Japan) with ATR MIRacle 10 was used to identify the functional groups. The spectra were obtained in the range between 4000 and 600 cm^-1^ with a resolution of 8 cm^-1^ by averaging 45 scans.

XRD analyzes were carried out on X’Pert MPD (Philips, Almelo, Netherlands) operating with step acceleration tension of 40 kV and electric current of 40 mA. The recording parameters were 5° min^-1^ and 2θ range from 10° to 75°. The diffractograms were analyzed using the HighScore Plus software.

Raman spectroscopy was performed on LabRAM Hr Evolution (Horiba Scientific) operating in 514,6 cm^-1^ and data analyzes through HighScore Plus software.

### Biotoxicity and assessment of cell viability analysis

Human bone marrow-derived mesenchymal stem cells (hBMSCs), specifically the iMSC3 line purchased from Applied Biological Materials (Richmond, BC, Canada), were used in the cytotoxicity assays performed in collaboration with UCLA (Regenerative Bioengineering and Repair Laboratory, Department of Surgery, David Geffen School of Medicine at UCLA), in accordance with ISO 10993-5 standards and in compliance with the Brazilian Health Regulatory Agency (Agência Nacional de Vigilância Sanitária – ANVISA) Resolution RDC No. 214/2018 and good manufacturing practices. Cells were initially seeded at a concentration of 1 × 10^5^ cells in each block composite. After seeding, the blocks incorporating hBMSCs were incubated at 37 °C for 2 h. Culture medium was then gently added to the wells (*n* = 3), performed in triplicate for each condition and time point. The blocks incorporating hBMSCs, along with the added culture medium, were then incubated further at 37 °C and 5% CO_2_ in a humidified incubator.

After 3 and 7 days of incubation, the blocks incorporating hBMSCs were transferred to a 48-well plate, and cell viability was assessed using the reagent WST-1, which is used to measure cell proliferation and viability.

WST-1 reagent (Millipore Sigma, Burlington, MA, USA) was added to the blocks incorporating hBMSCs and incubated for 1 h at 37 °C. Following incubation, the supernatant (the liquid above the cell block) from each well was transferred to a 96-well assay microplate. The absorbance of the supernatant was determined at 450 nm using a plate reader (SYNERGY H1, BioTek, Calabasas, CA, USA). The increase in absorbance observed on day 7 was calculated by subtracting the average absorbance measured on day 3 for each biomaterial.

### In vivo tests

The second step was to apply the scaffolds in an animal model with the aim of analyzing the bone repair process. Sixteen male Wistar rats (approximately 280 g) were obtained from Animal Vivarium of the Universidade Nove de Julho that approved all experimental procedures by the Ethics Council on Animal Use (Protocol no. 8675271021). All experimental procedures were performed in accordance with national guidelines (CONCEA and CEUA/UNINOVE) and regulations for the care and use of laboratory animals, and the study is reported in accordance with the ARRIVE guidelines. The experimental design, randomization, animal housing, and data reporting followed the essential recommendations of these guidelines. The rats were randomized and allocated (*N* = 4) into 4 groups. Group 1: CA; Group 2: CAG; Group 3: CAGO and Group 4: CANG.

The animals were anesthetized using a combination of ketamine and xilazine (80/10 mg/Kg) and two hind paws were prepared for surgery by trimming the hair. Subsequently, non-critical bone defects were created using a Driller™ implant motor (1.500 rpm) and a trephine drill with Ø1.3 mm with an access limiter coupled to the drill. All bone defects were filled with scaffolds previously prepared. Finally, muscle and skin were sutured and a combination of tramadol hydrochloride and dipyrone was administered intraperitoneally at a dose of 5 mg/kg for analgesia over a period of 3 days. On day 30 after surgery, all animals were euthanized with an anesthetic overdose for the purpose of tibia removal.

### Histological and histomorphometry analysis

The samples were decalcified, embedded in paraffin, and sectioned longitudinallyat a thickness of 0.5 μm. The sections were stained with Hematoxylin-Eosin (H.E.) and examined under a light microscope (Nikon Eclipse^®^ E400, Japan). Bone area quantification was performed by a single researcher blinded to group identification. Eight fields of the defect region were captured of each sample. Quantification for regenerated bone was performed using ImageJ (NIH, Bethesda, MD). The area of newly formed bone tissue and the total field area were measured. The ratio of bone formation area was calculated as the ratio of newly formed bone area to the total filed area.

### Statistical analysis

The data were expressed as mean ± standard deviation. The statistical significance was determined using ANOVA with alternative hypothesis (H_1_) with multiplePost Hoc comparison tests. It was considered significant p value < 0.05.

## Results

### Scaffolds microstructural characterization

**Fig. 1:**
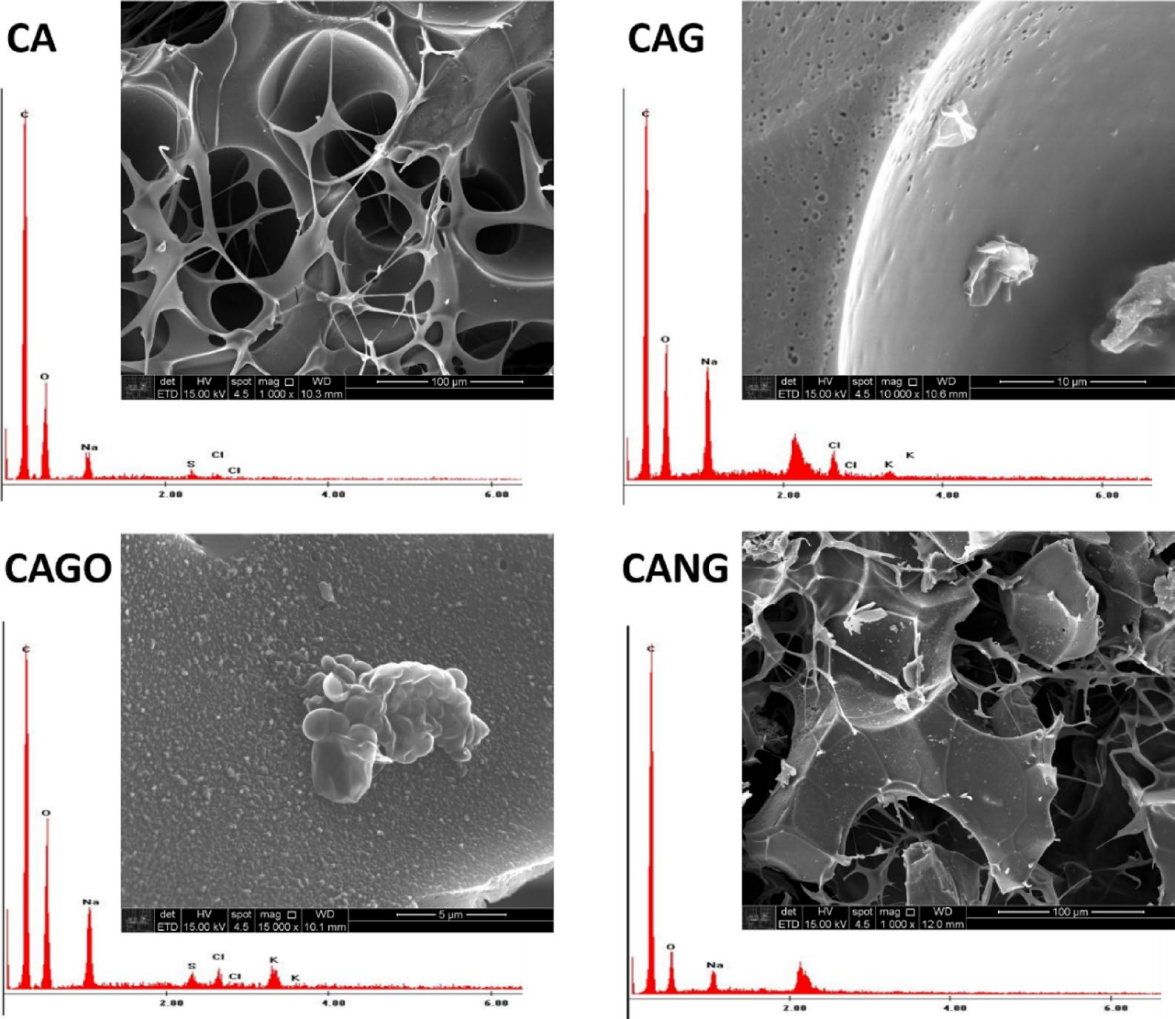
Chemical composition and SEM image of each type of Scaffold. Data obtained by energy-dispersive X-ray spectroscopy (EDS) –EDAX™ detector (122 eV; Ametek, NJ, USA).

The surface chemical composition was obtained by energy dispersive X-ray spectroscopy (EDS) and all of the compounds were corresponding to the materials expected for each scaffold (Fig. 1; Table 1). EDS analysis confirmed the expected chemical composition of the scaffolds, with carbon and oxygen as the main elements that are related with structural organization. CA, CAG, and CANG samples shows graphitic features with lower oxygen content, while CAGO exhibited the highest oxygen concentration, consistent with carboxyl and hydroxyl surface groups.

**Table 1:** Chemical composition and porosity detailing of carbon, oxygen percentage on material surface and surface area (S_BET_).

Samples	C (%)	O (%)	S_BET_ (m^2^/g^-1^)
CA	64,97	28,97	12,4035
CAG	57,86	29,14	14,7175
CAGO	53,58	33,99	12,9231
CANG	73,45	21,66	17,3226

The carbon–oxygen relationship suggests the presence of defects, as well as high hydrophilicity and surface energy, which influence cellular and protein adhesion. In addition, surface area and porosity are associated with nutrient diffusion, vascularization, and angiogenesis, all of which are essential for tissue growth.

#### Fourier transform infrared spectroscopy (FTIR) and Raman spectroscopy

**Fig. 2:**
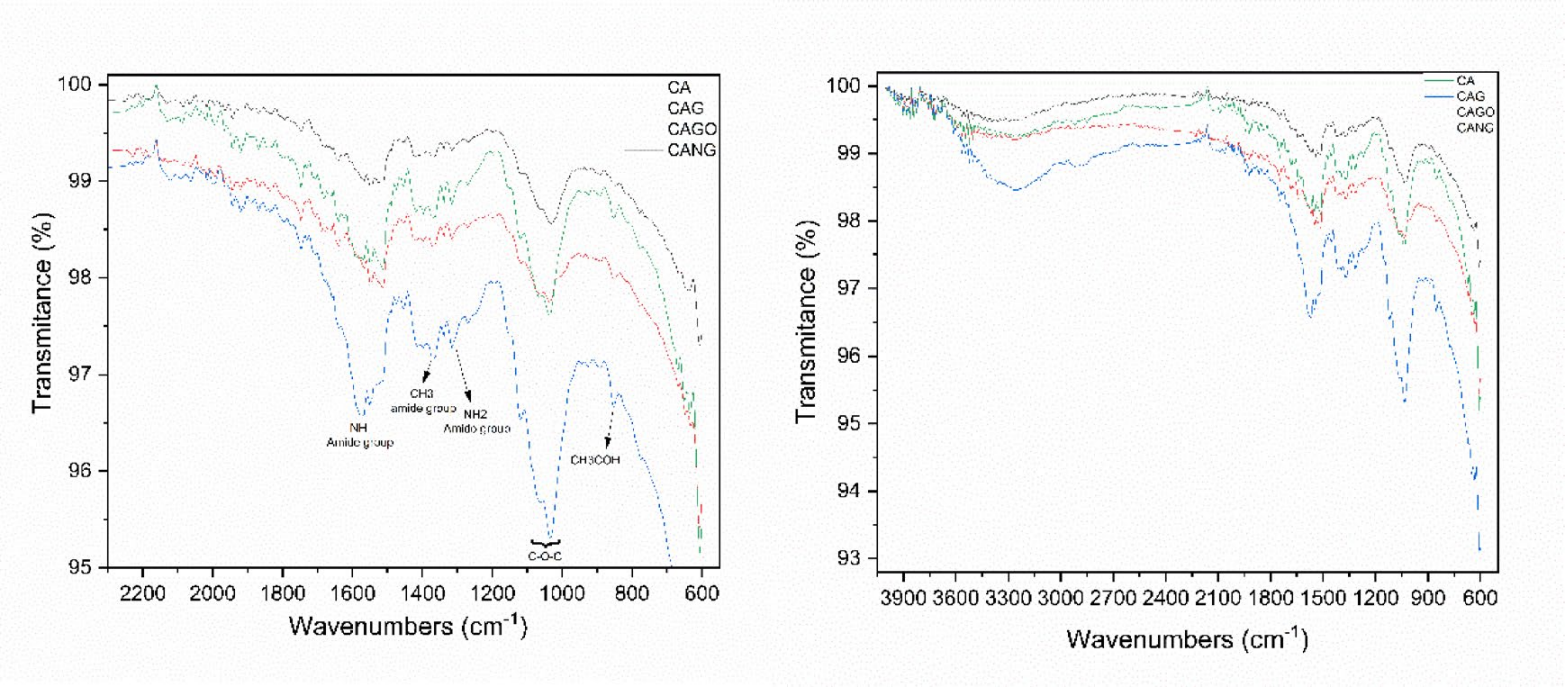
FTIR spectrum of scaffolds developed from carbon-related materials showing the transmittance and characterization of the functional group peaks founded in each sample.

The scaffolds produced in this study presented similar functional groups at 3260 cm⁻¹, consistent with O–H bonds; an intense peak at 1574 cm⁻¹ and a weak signal at 1370 cm⁻¹ corresponding to N–H and CH₃ amide groups, respectively. An NH₂ peak of the amino group was observed at 1313 cm⁻¹, and a double peak at 1119 cm⁻¹ and 1033 cm⁻¹ corresponds to C–O–C glycosidic linkages. Finally, a weak peak at 852 cm⁻¹ characteristic of CH₃COH was found (Figure 2).

The spectra revealed hydroxyl, carbonyl, and carboxyl groups. These oxygen-containing functionalities increase surface hydrophilicity, providing potential sites for protein adsorption and facilitating the initial biomaterial–cell interactions, which are crucial for subsequent cell adhesion and matrix deposition.

**Fig. 3:**
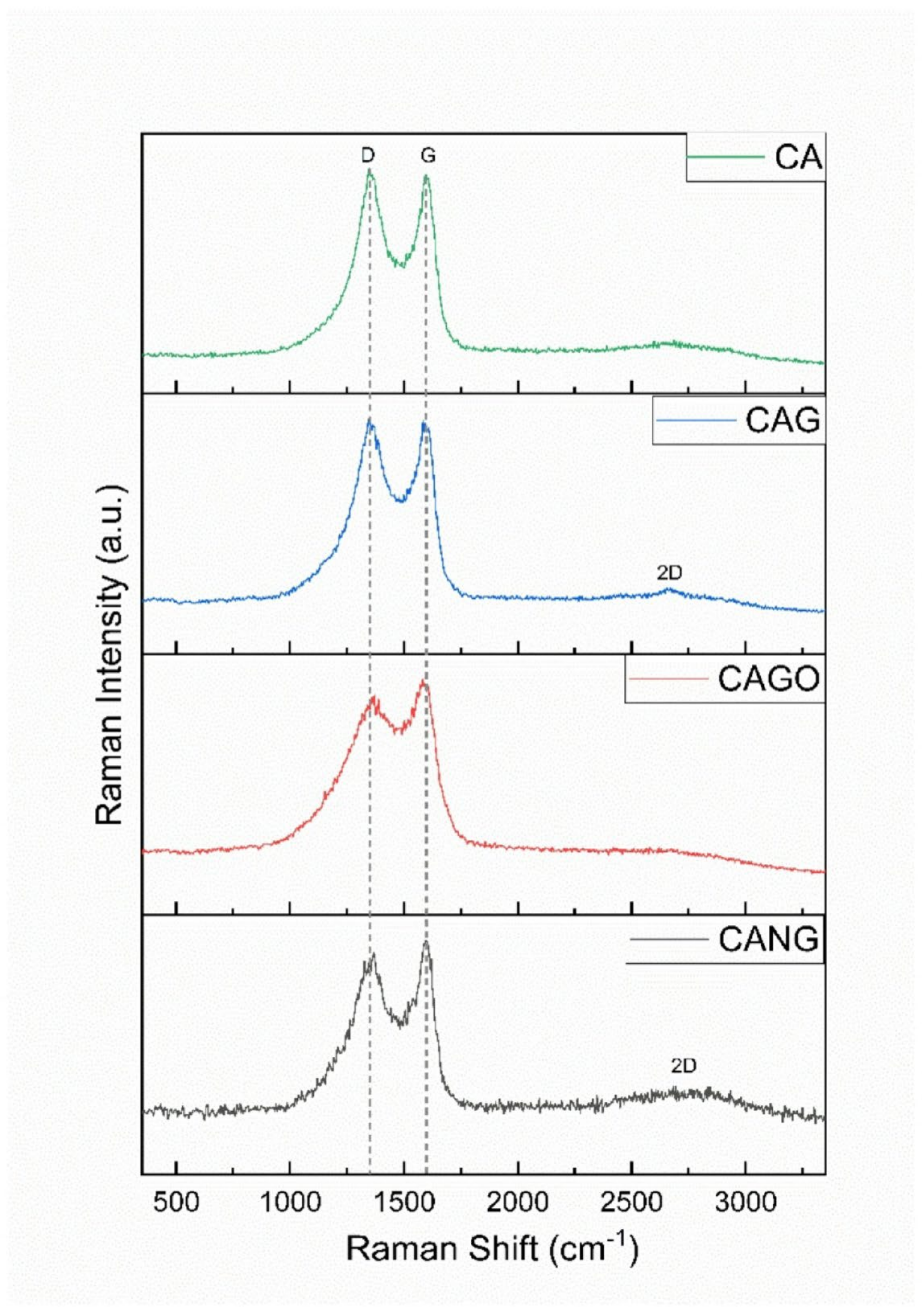
Raman spectra showing the D, G and 2D bands characteristic of amorphous carbon samples in all samples produced.

Raman spectrum analyses showed peaks characteristic of CA scaffold (D: 1347 cm⁻¹; G: 1595 cm⁻¹); CAG scaffolds (D: 1345 cm⁻¹; G: 1583 cm⁻¹; 2D: 2655 cm⁻¹); CAGO scaffolds (D: 1370 cm⁻¹; G: 1584 cm⁻¹); and CANG scaffolds (D: 1364 cm⁻¹; G: 1595 cm⁻¹; 2D: 2682 cm⁻¹), all evident in the samples. The observed 2D band peaks presented low intensities with broader and discrete peaks. D and G ratio intensity was similar across samples: CA 0.84; CAG 0.85; CAGO 0.86; CANG 0.85 (Fig. 3). All samples showed the characteristic D and G bands of carbon-based materials, with the 2D band appearing at low intensity. The D/G intensity ratio indicates a similar degree of structural disorder across the scaffolds.

#### X-ray diffraction (XRD)

**Fig. 4:**
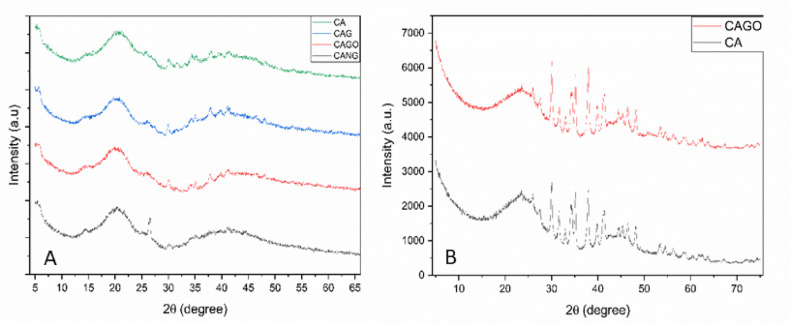
XRD diffractograms to all the samples. **A**) scaffolds containing chitosan-xanthan and carbon matrices; **B**) XRD pattern of carbon and carbon-graphene oxide powder.

XRD pattern analyzes is presented in Fig. 4 and shows reflections 2θ at 20° related to chitosan-xanthan to all scaffolds but can be also related to carbon nanoparticles used in this study. The reflections observed at 2θ = 5° correspond to surface defects and oxygen functional groups and the peaks around 26° to 45° are related with crystallinity of the materials. These diffraction data suggests that the carbon reflections were partially covered by the chitosan-xanthan signal present on the surface of the material. To confirm this affirmation, the carbon matrices powders were analyzed and show reflections between 30° and 50° characteristics of carbon materials (Fig. 4b). These data showed differences in crystallinity among the samples, with broader peaks indicating higher structural disorder. This observation is consistent with the EDS and BET analysis, as samples with higher oxygen and lower carbon proportions exhibited larger surface areas.

### In vitro biotoxicity testing and cell viability exposed to the biomaterials

All samples associated with Chitosan-Xanthan were organized as:

Group 1: (CA): bone defect grafted with Chitosan-Xanthan + Carbon;

Group 2: (CAG): bone defect grafted with Chitosan-Xanthan + Carbon + 0.1% of Graphene;

Group 3: (CAGO): bone defect grafted with Chitosan-Xanthan + 1% of Reduced Graphene Oxide in particles ≦ 250 μm;

Group 4: (CANG): bone defect grafted with Chitosan-Xanthan + Carbon + 1% of Nanografite.

Absorbance values ​​indicate the ability of different biomaterials to maintain cell viability. This characterization method allowed the interpretation of how each biomaterial supported cell survival. The experiments showed that all tested biomaterials led to an increase in cell viability after 4 days of incubation.

Statistical analysis was performed using the Kruskal-Wallis test and Dunn’s multiple test finding the absence of a statistically significant difference between the tested groups.

**Fig. 5:**
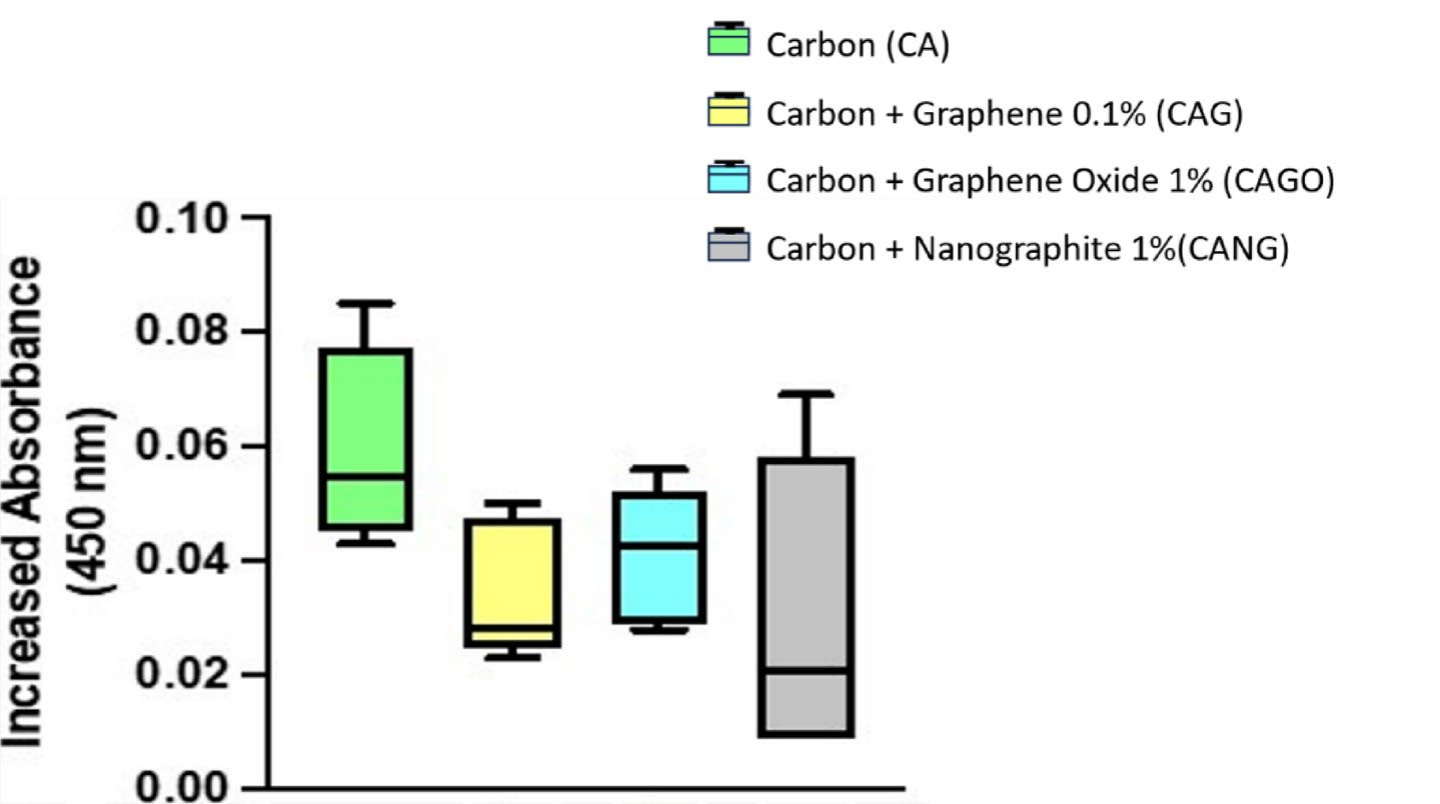
Boxplot chart showing the increased absorbance of all biomaterials tested, indicating the capacity to maintain cell viability.

Based on the results of absorbance, all tested biomaterials (Carbon, Carbon + Graphene 0.1%, Carbon + Graphene Oxide 1% and Carbon + Nanographite 1%) were found to be supportive to cell survival (Fig. 5) and, consequently, non-cytotoxic.

### Histological and histomorphometry analysis

Taking the same group organization as in in vitro tests, histomorphometry assessment was carried out using histological cross sections, from the samples of 16 Wistar Rats tibial defects, stained in Hematoxylin Eosin (HE) with ten times (10x) magnification.

**Fig. 6:**
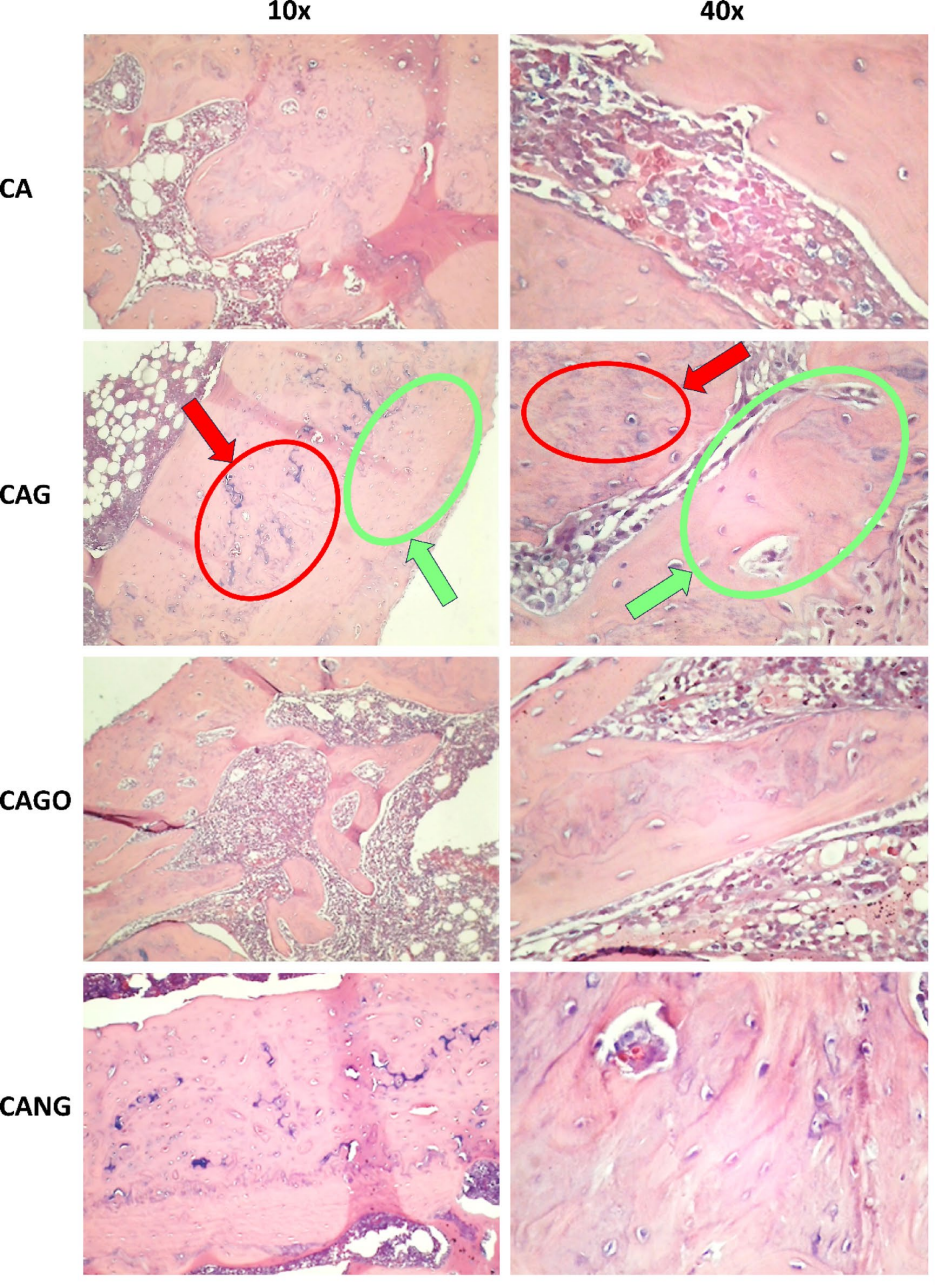
Histological optical microscope cross-sections of the implanted biomaterial in Wistar Rats tibial defect. Optical microscopy images by groups (rows) and the magnifications (columns). The red arrows indicate the presence of unabsorbed graphene debris, osteoblasts, osteocytes and capillaries within the immature newly formed bone. The green arrows indicate more mature bone with vascularization and osteocytes in more restricted gaps without the presence of graphene. Both are indicated in 10 and 40x magnification of CAG Group cross sections.

Each group was composed of 4 animals (8 samples obtained) and had implanted the respective biomaterial. Examples of the cross sections used in the histomorphometry assessment are elucidated in Fig. 6.

**Table 2:**
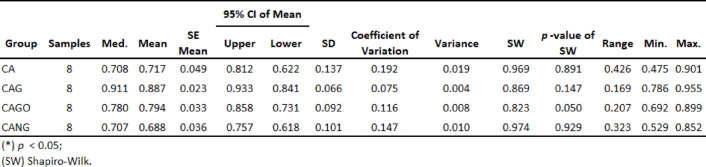
Descriptive Analysis with each group Confidence Interval (CI), Standard Deviation (SD) and Variance. Shapiro-Wilk check confirmed groups values homogeneity.

**Fig. 7:**
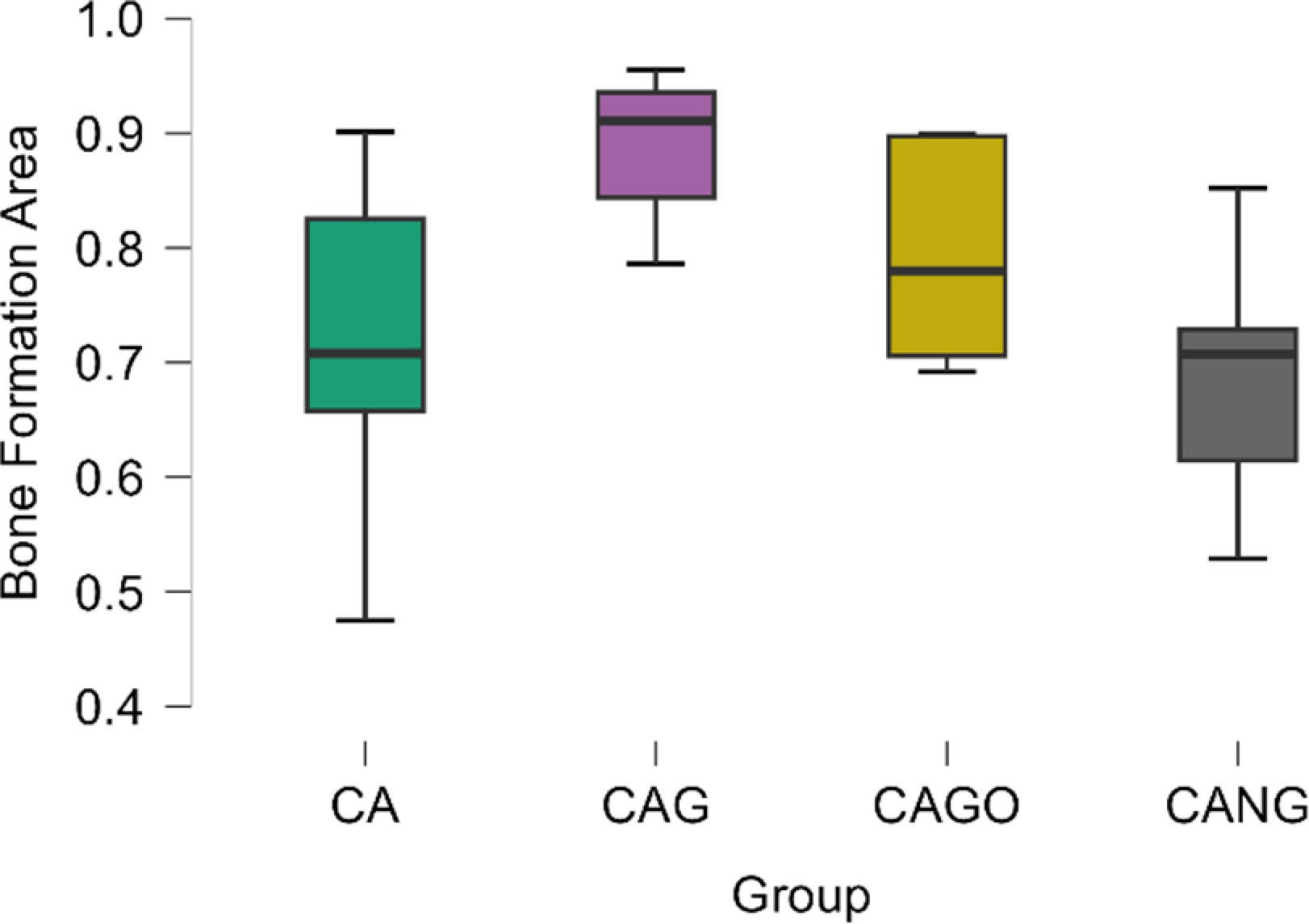
Boxplots chart of the mean value of each group, 95% Confidence Interval and Standard Deviation showing a superiority of bone formation area in absolute numbers.

Among the biomaterials used, the average amount of bone repair varied between the groups, from 69% (CANG) to 89% (CAG) of new bone formation in 30 days, as it can be seen in Table 2 and Boxplot below (Fig. 7).

Three homogeneity ANOVA corrections were applied and resulting in significant with *p* < 0.01 (Table 3). Also, Levene´s test for Equality of Variances result in no significant *p*-value among the groups studied (Table 4).

**Table 3:**
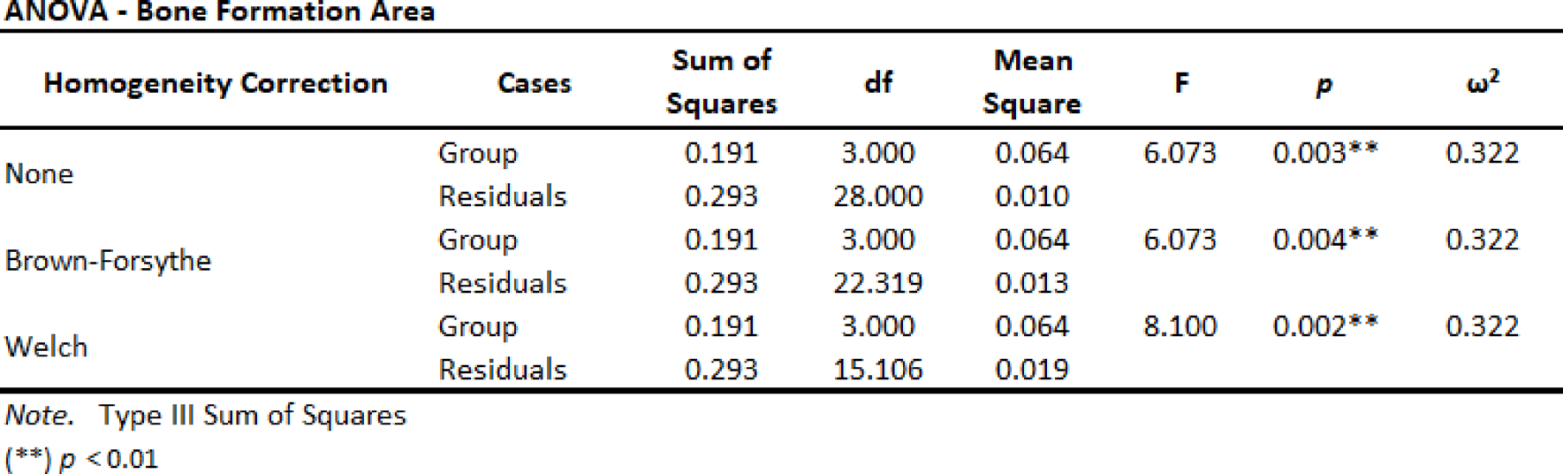
Homogeneity correction Sum of Squares and residuals.

**Table 4:**
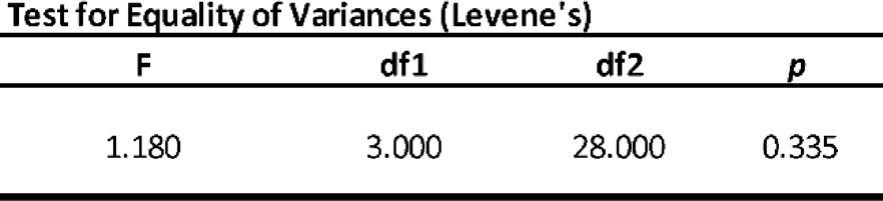
Test for Equality of Variances.

Both, ANOVA Homogeneity Corrections and Levene´s test showed positive Factors tests. These results provide the basis for ANOVA Tests of Contrast between groups (Tables 5 and 6) and Post-Hoc Comparison Tests (Tables 7, 8 and 9).

**Table 5:**
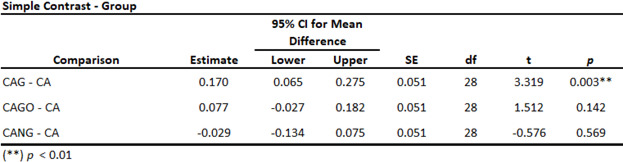
ANOVA Simple Contrast Test with control Group (CA).

**Table 6:**
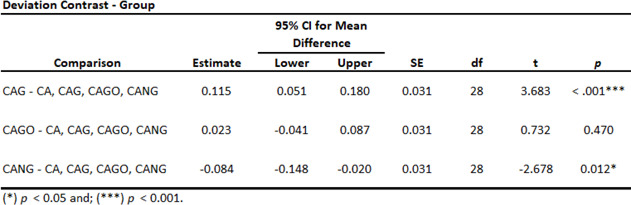
ANOVA Deviation Contrast Test.

**Table 7:**
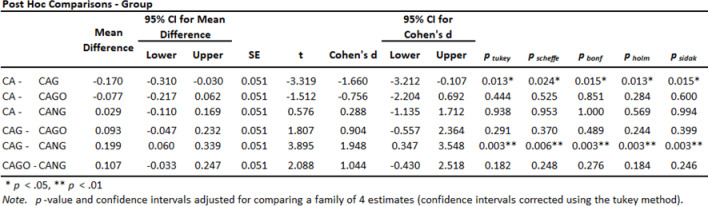
Standard Post Hoc Comparison Tests.

**Table 8:**
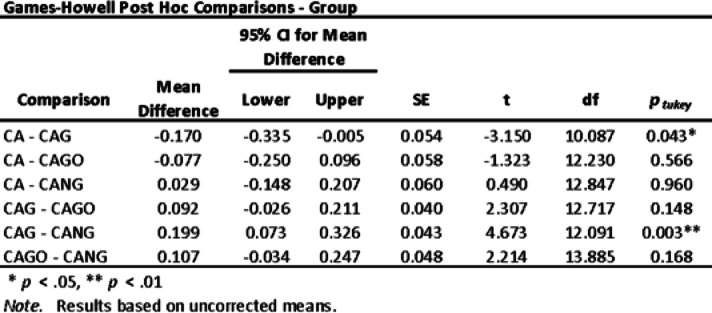
Games-Howell Post Hoc Comparisons test.

**Table 9:**
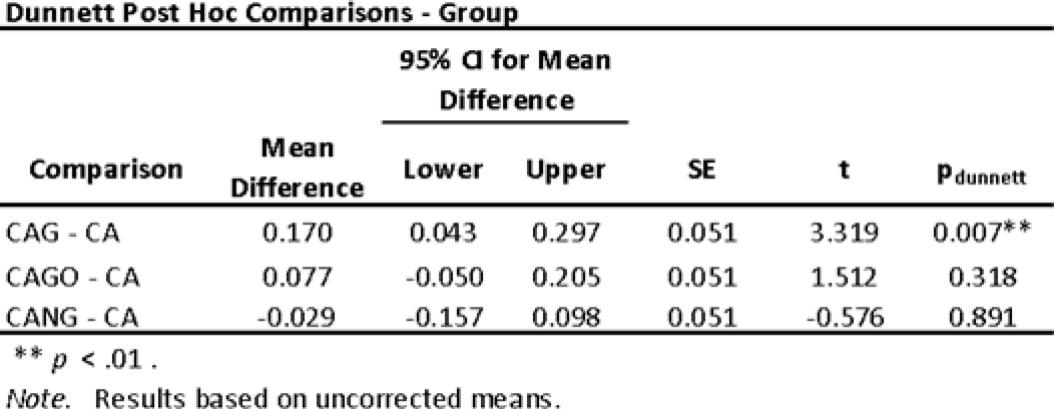
Dunnett´s Post Hoc Comparisons Test.

**Fig. 8:**
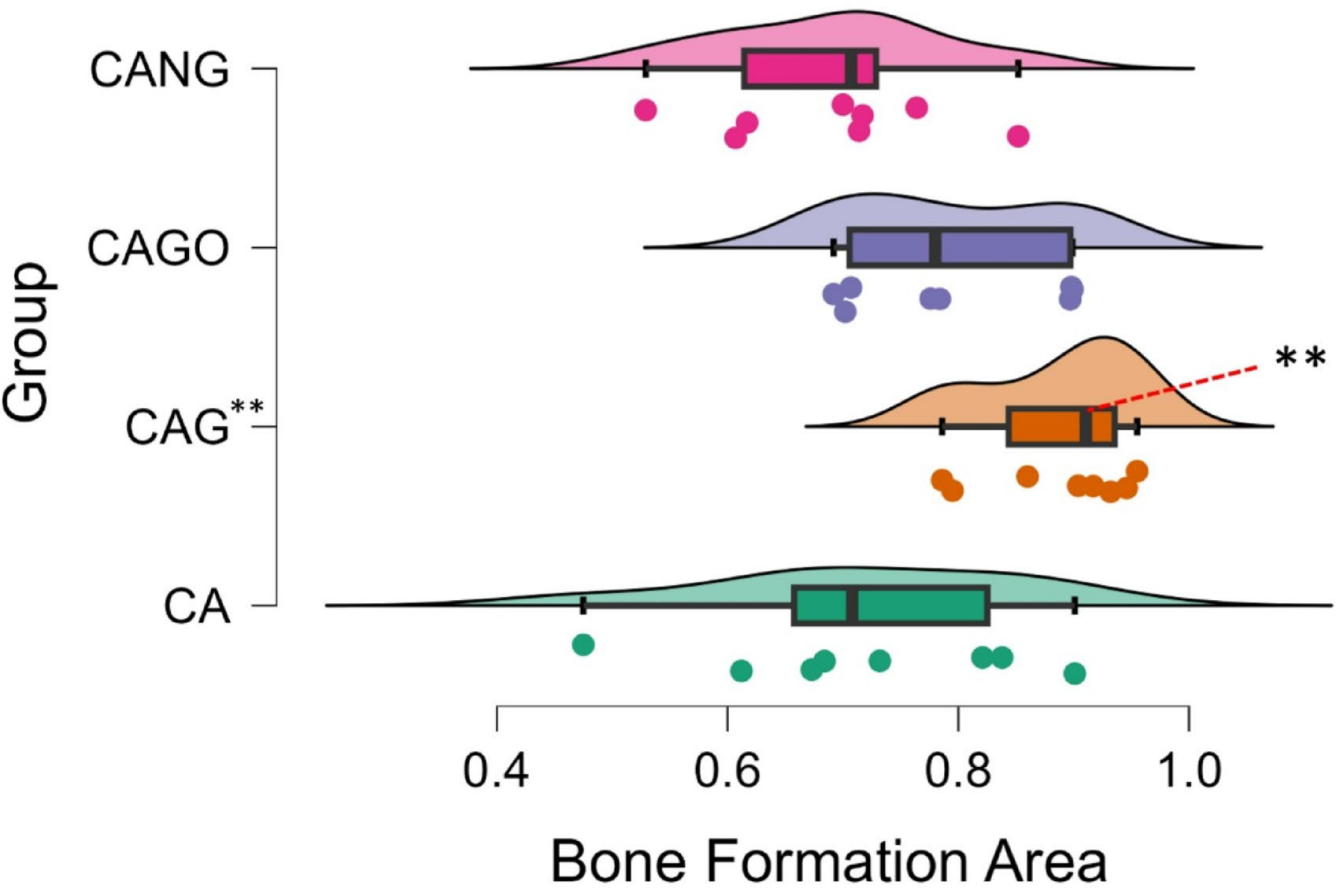
Raincloud plots. Bone Formation Area Average (in absolute numbers) considering the data obtained in Histomorphometry Analysis with 10x magnification and Comparison of Bone Formation Ratio by each biomaterial. (**) *p*-value < 0.01 statistically significant Dunnett´s Post Hoc Comparison Test with Control Group (CA).

The experiments carried out on rats, collected data by Histomorphometry and ANOVA statistical analysis, demonstrated that Groups 1 (CA), 3 (CAGO) and 4 (CANG) are statistically similar with 95% confidence for comparing averages, but the Group 2 (CAG) with Chitosan-Xanthan + Carbon + 0.1% of Graphene had a statistically significant difference to Group 1 (CA) with Chitosan-Xanthan + Carbon in Dunnett´s Post Hoc Test (*p*-value < 0.01) and at least a difference of *p*-value < 0,05 to the other Groups in the other Post Hoc Comparison Tests. Also, CAG presented the lowest standard deviation (89 ± 6% of bone area formation) following statistical analysis (Fig. 8).

## Discussion

Carbon and their derivatives have been studied for many years for medical applications, and more recently, in the multidisciplinary field of tissue engineering^9,29^.

Due to the carbon physicochemical characteristics, the diversity of protocols for using carbon in biomaterials for bone tissue engineering, the new functionalization technologies, and the numerous positive effects of using these elements in in vivo and in vitro studies, these materials have increasingly gained prominence in translational research studies^30^. In this study, we observed the possibility of developing a three-dimensional carbon-graphene-based biomaterial with biocompatible properties that was capable of promoting adequate bone tissue growth “in vivo” without post-implant complications, corroborating with other studies in the literature^31,32^.

In this study, the carbon was obtained directly from crude black liquor, which makes the production process of the biomaterial cheaper and faster than that observed by other researchers^33,34^. Furthermore, a superficial analysis of the chemical composition via EDS (Table 1) showed a high carbon yield, a large amount of oxygen present in all scaffolds, and the absence of potentially toxic elements (Fig. 1) that were eliminated during synthesis. This indicates the efficiency of the method used for obtaining our carbon biomaterials, which are safe for biological applications. These findings suggest that the methodology used in this study can be an alternative route to the protocols of other works^34–36^. Here it demonstrates the advantages of using black liquor as a precursor for carbon materials due to the abundance of this industrial waste and the low acquisition cost, eliminating multiple purification steps that increase time, cost, and affect yield.

It was observed that the uniform microstructure exhibited pore interconnectivity, adherence of the polymeric matrix to the carbon particles, and the presence of graphene and graphene oxide nanomaterials on the surface (Fig. 1). The physicochemical characteristics are fundamental in the development of scaffolds for regenerative medicine, since cellular survival depends on the unrestricted supply of nutrients and oxygen^37^, which flow through the available pores and are related to the mineralization of bone tissue. Our SEM and BET analyses demonstrated interconnected porosity and surface area suitable for cell adhesion and proliferation, consistent with findings by Akbari-Aghdam et al. (2021)^38^, who reported 55–61% porosity in HA-SWCNT scaffolds produced by DLP 3D printing. This similarity reinforces the importance of controlled pore size and distribution to support nutrient diffusion and bone tissue growth in engineered scaffolds. Moreover, high porosity is also related to the available surface area and intracellular cascades for cellular adhesion and colonization, ensuring not only survival but also angiogenesis and enabling new tissue formation^39–41^.

Our structural analysis also observed a surface area and pore size that were relatively small compared to other similar carbon-related materials^42,43^. Our scaffolds were fabricated with the addition of PMMA as a sacrificial structure to increase the pore formation, but the addition of chitosan-xanthan to the protocol for particle aggregation and scaffold malleability compromised the calculation of total pore volume and its type determination. The data from the surface and porosity analysis of the scaffolds raise the possibility that the polymeric compound may have caused pore blockage in the carbon matrix, as without this composition the carbons showed surface area, volume, and pore size considerably higher^6,44^. Fahim et al. (2015)^45^ observed that chitosan associated with graphene and fullerene nanofillers causes an increase in pore blockage and a reduction in size. Salehi & Farahani (2017)^46^ observed a similar polymer membrane on the carbon surface and a reduction of material porosity. Despite the porosity data obtained in our study, tissue growth was not compromised, as observed in histomorphometry analyses and suggests that even with low porosity, the scaffolds developed in this study may benefit tissue repair, and this aspect may be improved by the possible addition of functional groups on the material surface.

The FTIR analysis showed that all scaffolds exhibit similar functional groups, with an O-H bond at 3260 cm^− 1^; a peak at 1574 cm^− 1^ attributed to graphene (C = C vibration); and a peak at 1370 cm^− 1^ related to N-H and CH_3_ groups from the chitosan-xanthan structure. An NH_2_ peak of the amino group at 1313 cm^− 1^ and 1119 cm^− 1^ can be related to interactions between the chitosan-xanthan and carbon matrices via amide and carboxylic groups, while the 1033 cm^− 1^ peak (C-O-C, epoxy or alkoxy) is attributed to graphene oxide bands. One peak at 852 cm^− 1^ corresponds to the C-H group. Rezaei et al. (2021)^47^ correlate these peaks, and their shifts in position and intensity, with interactions between carbon nanomaterials and the polymer matrix. Our results also corroborate findings from Drewniak et al. (2016)^48^, showing similar vibration patterns of carbon nanoparticles, which are consistent with the observed biocompatibility reported by Zhu et al. (2021)^49^, Smolka et al. (2021)^50^, and Liu et al. (2021)^51^, suggesting that these scaffolds possess functional groups favorable to adhesion, proliferation, and cellular growth.

XRD analysis revealed that the scaffolds are composed mainly of amorphous components, consistent with previous reports^52^ and with the carbon derived from lignin present in black liquor^6,53^. The diffraction peak at 2θ = 14° is related to the oxidation of graphite, whereas the peak at 2θ = 26° reflects graphite crystallinity and high structural surface organization. The fabrication protocol aims to enhance the physicochemical properties of these materials for medical applications.

Raman spectra showed characteristic peaks of carbon-related materials, with C-C bonding vibrations in the sp² configuration and structural disorder typical of some carbon nanomaterials^52,54–56^. These data provide insights into surface and defect characteristics, which can be exploited for functionalization or molecular delivery to enhance bone repair. These carbon-related scaffolds have potential in bone tissue engineering due to their surface functional groups, porosity, and structural features, which promote protein adsorption, cell adhesion, proliferation, and osteogenic differentiation. Their structural framework and mechanical properties also support tissue integration and load-bearing applications, making them promising for accelerating bone regeneration and improving the mechanical strength of the repaired bone and scaffold.

Finally, the chemical composition of black liquor critically influences the resulting carbon-based biomaterials. Carbon content determines the scaffold’s structural framework and functional group density, enhancing protein adsorption and cell interactions. Phenolic and aromatic compounds contribute to structural integrity and mechanical strength during processing. Together, these chemical features modulate surface functionalization, porosity, and structural disorder^53,55^, which are essential for cell adhesion, proliferation, and extracellular matrix deposition, supporting the scaffold’s suitability for bone tissue engineering.

Despite their promising properties, several challenges must be addressed to ensure the effective application of black liquor-derived biomaterials in orthopedic settings. These include ensuring uniform dispersion of carbon and graphene within the scaffold and optimizing processing parameters to achieve reproducible porosity and mechanical strength^38^. Additionally, strategies to prevent potential cytotoxicity, manage structural defects, and maintain scaffold stability during implantation are critical for translating these materials into clinical use.

In addition to the structural results, the cytotoxicity analysis of the biomaterials developed by our research group demonstrated an increase in cell viability of stem cells (hBMSCs) in all scaffolds, after 4 days of incubation, showing that they are biocompatible and corroborating with the findings of the literature^56–59^. Graphene synthesized by chemical deposition was used by Nayak et al. (2011)^13^ to investigate the cell toxicity and physiology changes caused by this nanoparticle seeded with stem cells. The researchers observed that graphene promoted acceleration of hMSCs differentiation process with no changes in cell function or morphology. Saravanan et al. (2017)^55^ observed an increase in osteoblast differentiation with graphene-chitosan scaffolds in stem cell culture, and upregulation of gene markers.

Carbon-related materials have been used in biological applications for a long time; however, the data about the influence of functional surface groups in bone tissue engineering are diverse^60,61^, and this can be explained by differences in carbon composition, precursors, and concentrations in the biomaterial formulation. Our results coincide with findings from various authors^62–67^. who attest the benefits of using carbon, graphene, graphene oxide, and other carbon allotropes in medicine and bone tissue bioengineering^29^.

The analysis of bone formation results in an animal model showed that all scaffolds developed by our research group were capable of promoting bone repair of the defect created in the rats’ tibias, with approximate percentages of newly formed bone between the groups 30 days after the implant. The presence of osteoblasts and large areas with medullary bone tissue were observed regardless of the composition of the biomaterials, and the Carbon Graphene 0.1% group showed the highest percentage of bone formation compared to the others. This data was confirmed by histological findings that showed an extensive area of new bone formation, a large number of osteoprogenitor cells, and a more organized bone tissue. Macroscopically, all bone defects “in vivo” were completely repaired at the end of the experimental period, and despite the presence of carbon particles in some histological sections, no alterations in tissue repair were observed where these particles were found. The literature shows that the bone repair capability of other biomaterials, such as composites of hydroxyapatite and collagen reinforced by carbon nanotubes, promotes the improvement of the mechanical aspects of the biomaterials and provides an increase in bone tissue repair after the implant^68^. Similar results were observed when a biomaterial composed of sodium hyaluronate with graphene oxide demonstrated that the presence of graphene promoted accelerated bone repair without cellular damage^69^.

The data obtained in the present study corroborate those findings from the literature that demonstrate that carbon-related materials enhance bone repair and present themselves as an excellent option as biomaterials for application in bone tissue bioengineering. However, the observation of some controversial results suggests that further studies should be conducted to elucidate and ensure the benefits of using these nanomaterials in clinical practice^29,30^.

It is worth noting that various areas of medicine and dentistry can benefit from the development of new biomaterials containing carbon-graphene; however, these biomaterials should be developed with appropriate concentrations and associated with other biomaterials according to the particularities of the clinical manifestation of each bone pathology to be treated by tissue bioengineering. One of the key motivations for the development of the present study is related to the problems faced by orofacial surgeons in treating cleft patients who suffer from the low specificity and high cost of the materials available on the market for use in bone tissue engineering. Another motivation is the potential for utilizing industrial waste to produce these carbon-graphene-based biomaterials, opening a new production route that favors the productive ecosystem and the environment.

Despite the promising results obtained in this study, some issues will need to be addressed later, such as the lack of details on gene expression correlated with bone formation according to the concentration of carbon-graphene used for the biomaterial production and a detailed analysis of the material porosity. Another point to be addressed in the future is the colonization of these scaffolds with mesenchymal stem cells to evaluate the possibility of obtaining 3D bone growth as well as to study the potential use of carbon-graphene for formulating bioinks that allow the printing of bone tissue scaffolds in different shapes according to each pathology.

## Conclusion

This study demonstrated that biocompatible carbon-graphene biomaterials for bone tissue engineering can be effectively fabricated from black liquor. Independent of the graphene concentration, consistent bone formation was observed in vivo. Notably, at a concentration of 0.1%, newly formed bone exhibited superior histological quality 30 days after biomaterial implantation. These findings open new avenues for the development of graphene–carbon scaffolds for bone tissue engineering. Beyond their sustainable origin, black liquor–derived scaffolds displayed distinctive biological features—such as surface functionalization, controlled porosity, and inherent bioactivity—that differentiate them from conventional carbon-based biomaterials and reinforce their suitability for clinical application in bone repair. Furthermore, this innovative production strategy not only contributes to a circular and sustainable ecosystem but also significantly reduces manufacturing costs, thereby offering both environmental and economic benefits.

## Data Availability

As this is an ongoing research project, all datasets generated and/or analyzed in this study are available from corresponding author upon reasonable request.
